# Intelligent Spectroscopy System Used for Physicochemical Variables Estimation in Sugar Cane Soils

**DOI:** 10.3390/s19020240

**Published:** 2019-01-10

**Authors:** Ofelia Landeta-Escamilla, Oscar Sandoval-Gonzalez, Albino Martínez-Sibaja, José de Jesús Agustín Flores-Cuautle, Rubén Posada-Gómez, Alejandro Alvarado-Lassman

**Affiliations:** 1 Tecnológico Nacional de Mexico/I.T.Orizaba, Orizaba, VZ 94320, Mexico; of_elia@hotmail.com (O.L.-E.); o.sandovalgonzalez@gmail.com (O.S.-G.); pgruben@yahoo.com (R.P.-G.); lassman@prodigy.net.mx (A.A.-L.); 2 CONACYT-Tecnológico Nacional de Mexico/I.T.Orizaba, Orizaba, VZ 94320, Mexico; jflores_cuautle@hotmail.com

**Keywords:** psychochemical prediction, frequency response of soil, FPGA-based

## Abstract

The current condition of soils is a major area of interest due to the lack of certainty in their physicochemical properties, which can guarantee the quality and the production of a specific crop. Additionally, methodologies to improve land management must be implemented in order to address the consequences of many environmental issues. To date, many techniques have been implemented to improve the accuracy—and more recently the speed—of analysis, in order to obtain results while in the field. Among those, Near Infrared (NIR) spectroscopy has been widely used to achieve the objectives mentioned above. Nevertheless, it requires particular knowledge, and the cost might be high for farmers who own the fields and crops. Thus, the present work uses a system that implements capacitance spectroscopy plus artificial intelligence algorithms to estimate the physicochemical variables of soil used to grow sugar cane. The device uses the frequency response of the soil to determine its magnitude and phase values, which are used by artificial intelligence algorithms that are capable of estimating the soil properties. The obtained results show errors below 8% in the estimation of the variables compared to the analysis results of the soil in laboratories. Additionally, it is a portable system, with low cost, that is easy to use and could be implemented to test other types of soils after evaluating the necessary algorithms or proposing alternatives to restore soil properties.

## 1. Introduction

A global issue to attend due to its relevancy is soil degradation: firstly, because it is a natural resource that is formed and regenerated slowly, is not renewable, and is very fragile in its degradation [1]. Therefore, it is one of the most vulnerable natural resources, because it is not possible to take actions to avoid or mitigate the adverse impacts that are produced by environmental detriment [2]. Soil plays a crucial role in the functioning of ecosystems [3] due to the impact on agronomics and food security [4], by decreasing its current and potential capability to produce goods and services. Land management is far from being sustainable; there has been an inadequate operation of soils in the conventional production systems [5]. For example, the excessive use of fertilizers has led to nitrogen depositions affecting not only surface and underground water, but also soils with acidification, because an incorrect application of modern agrotechnologies [6] generates unstable ecosystems, which in turn creates an external dependence on energy and materials for their continuity in time [7]. In turn, this increases the number of limiting factors in the production systems in relation to the nutrients and water, interfering in the biogeochemical cycles [8]. In contrast, there are also places where the lack of fertilizers is degrading soils and decreasing yield, which is important because they also help with carbon fixation to mitigate climate change. Hence, it is necessary to monitor the health of soils [9] to determine their current status, and also to monitor nutrient levels in order to apply sustainable land management practices.

There are many approaches to determine the level of soil degradation. Some of them treat the problem as a single one instead of a complex process, integrating variables such as: vegetation growth, the flow of water, infiltration, land use, and land management. Furthermore, some variables could be partially hidden by management practices, the use of fertilizers [10], and land resilience [11]. Some others analyzed information from global datasets, which may mask local changes; it is important to remember that most relationships are based on local studies, and should not be used to infer global conclusions.

A more detailed physicochemical analysis by methodologies carried out at laboratories requires glassware, apparatus, chemicals, and trained personnel. Although these methodologies have been validated, in order to assure quality control, it is necessary to have controlled samples to evaluate method precision and uncertainty. That makes the access difficult for farmers, as not all of the samples that are taken are representative of the real condition of soils, and some locations are impossible to access.

Thus, investigations have been carried out in order to obtain correct results in the field with the use of different sensors and technology, reducing the time and cost of the regular analysis. Sensors operate by measuring physical or chemical properties via transducers, while another technology has focused on linking sensors with artificial intelligence or satellite images to infer the soils’ situation in general.

Methods to determine chemical properties include: (1) electrical/electromagnetic sensors to obtain organic matter (OM) or total carbon content, salinity, cation exchange capacity (CEC), and residual nitrate or total nitrogen content; (2) optical and radiometric sensors to determine OM or organic carbon (OC), pH, CEC, residual nitrogen or total nitrogen content; and (3) electrochemical to determine salinity, pH, residual nitrate or total nitrogen content, and other macronutrients [12]. The property used in electrical and electromagnetic sensors is the electrical conductivity (ECa). Accordingly, ECa data electromagnetic interference sensors (EMI) can be linked to a global positioning system (GPS). The literature reports contain soil properties and pasture yield results collected over four years, finding significant variations in macronutrients (50–110%) with 20% to 26% of the variability in OM and clay content and stability (less than 10%) for pH and ECa throughout the area evaluated. Significant correlations have been found between ECa, altitude, and pH; correlations between ECa, grasses, yield, and other species were also found. Despite the correlations already reported in the literature, there is still a controversy. While Serrano et al. [13] concluded that no correlation was found, Kweon et al. determined an excellent correlation when a second chemical variable (CEC) is included; this highlights the importance of using a matrix of variables to achieve reliable results.

Another pathway that has been implemented to determine chemical properties is optics, where spectroscopy is used due to its sensitivity [14]. Methods relay the comparison of the incident and reflected light spectra on soils, using the amplitude and phase of signals [15]. This technique has been implemented in several studies to determine many variables; such as moisture, OM, pH, EC, CEC, TC, ammonium, nitrogen, nitrates, total nitrogen (TN), available phosphorus, and phosphorus absorptive coefficient (PAC), where 12 spectroscopic models were developed and the correlations that were obtained were in the range of 0.45 to 0.93 [16].

Spectroscopy has been used along with a wireless sensors network (WSN) for improving the measurements’ precision, monitoring variables such as irrigation, fertilization, pesticide control, and animals in large areas [17]. All of those variables are being monitored using the normalized soil moisture index (NMSI) obtained from reflectance values and exploratory analysis of the raw spectra by using an artificial intelligence tool, the principal component analyses (PCA) [18]. On the other hand, an impedance spectroscopic sensor is capable of measuring multiple frequencies to determine soil moisture and ionic content, and has a built-in self-calibration system. An advantage of these systems is the capability of calibrating the system response to different soil types. Furthermore, their main disadvantage is the presence of noise, which results in decreased accuracy. Results report having a 10% error range for real and imaginary impedance and 12% for soil saline water content measure, which enables mixed models to determine specific ions such as nitrates, sulfates, and phosphates.

From previous lines, it stands out that grouping technologies promises to be a suitable solution to determine the level of degradation in soils accurately, and thus propose local solutions based on the causes found at the specific locations. Additionally, the relationship between poverty and the percentage of land degradation is also a separate issue, because it highlights the need to propose new low-cost technology in order to determine the needs at these places and promote sustainable land management practices. Therefore, a sensor that is capable of determining physicochemical properties through artificial intelligence techniques could create a low-cost and easy to implement technology. If farmers could operate it, such a sensor would create technology that can know the current state of soils and enable the implementation of restoration strategies.

## 2. Materials and Methods

### 2.1. Soil Sampling

The region of so-called high mountains in Veracruz, Mexico is formed by 57 municipalities with an approximate area of 6053 km^2^. The 0.5% of the total surface is planted by sugar cane; the studied soil can be classified as anthrosols (AT) according to World Soil Resources [19]. Therefore, an essential point to develop the presented system is the sampling of soils with sugar cane production made in high mountain regions. One hundred soil samples along this mentioned area were obtained, and the sampling procedure implemented to obtain the soil samples was the one described in the standard SESDPROC-300-R3 [20]. Specifically, the obtained soil samples were collected at the municipalities of Atoyac (18°55′00″ N 96°46′00″ O), Camaron de Tejeda (19°01′00″ N 96°37′00″ O), Carrillo Puerto (18°47′00″ N 96°34′00″ O), Coetzala (18°47′00″ N 96°55′00″ O), Ixtaczoquitlan (18°51′46″ N 97°03′44″ O), Cordoba (18°51′18.3″ N 96°57′02.2𠌽 W), and El Naranjal (18°47′38.1″N 96°55′30.5𠌽 W), which are areas that traditionally cultivate sugarcane. All of the samples were subject to laboratory analysis to obtain the following physicochemical properties: pH, tampon pH, organic matter (OM), phosphorus (P), Potassium (K), Calcium (Ca), Magnesium (Mg), Sulfur (S), Boro (B), Copper (Cu), iron (Fe), Manganese (Mn), Zinc (Zn), Sodium (Na), conductivity, Nitrogen–Nitrate (N_2_–NO_3_), cation exchange capacity (CEC), cationic saturation (SC) for K, Ca, Mg, and Na, Hydrogen (H), K/Mg ratio, Ca/Mg ratio, texture (sand, lime, and clay), apparent density, field capacity 1/3 bar, and permanent wilting point, 15 Bar.

### 2.2. Physicochemical Analysis

Sample moisture and the OM to soil moisture ratio was determined by gravimetry [21]. The humidity was obtained by weighing the sample and heating it to 105 °C for 24 h until all of the water was lost at a constant weight. The difference between the wet and dry sample gives the moisture content.

The Walkley and Black method [22] was conducted with the aim of determining the amount of organic matter (OM) expressed regarding total organic carbon.

### 2.3. Chemical Composition

Atomic absorption spectrometry was used to determine the global composition of Na, K, Mg, and Ca in soils. The composites were digested in hot HCl and deionized distilled water solution (2:1 ratio); afterward, the solution was filtered and submitted for analysis.

### 2.4. Exchangeable Cations, Nitrogen, Phosphorous, and Sulfur

Exchangeable cations (CEC) were measured using silver thiourea, following the method described by Pleyser and Juo [23]. Total nitrogen was measured by the Kjheldhal method [24], phosphorous was measured by colorimetry [25], and sulfur was measured by turbidimetry [26]. Apparent density was measured by the method proposed by the United States Department of Agriculture (USDA) [27], pH was measured by the 1:1 method ASTM D4972-13 (ASTM 2013), and electrical conductivity (EC) was measured by the conductimetry method. The textural analysis was performed by the Bouyoucos method.

### 2.5. Measurement Principle

**Figure 1:**
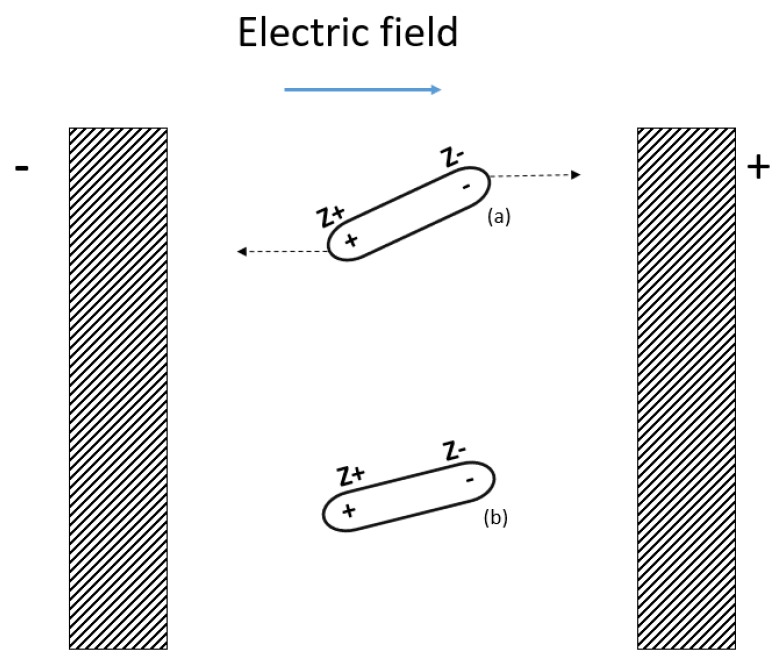
Molecule polarization under the electric field influence (**a**) original state, (**b**) polarized.

When an electrical isolating and homogeneous material is under the influence of an electrical field (*E*), each molecule forming the material is affected by the field, as shown in Figure 1. As a result, all of the molecules are subject to an electrical force, and an electric dipole is formed. Forces acting on the dipoles are named electric momentum (*µ_i_*), whose value is proportional to the electrical charge (*z*) and the distance between electrodes (*l*):

(1) $$\mu_{i}=z\times l$$

The electrical momentum forces the molecules to rotate; then, the dielectric displacement is expressed as:

(2) $$D=\varepsilon \times E+P_{i}$$

where ε is the dielectric constant, and *P_i_* is the induced material polarization for the *i*st molecule, the Clausius–Mossotti equation establishes:

(3) $$P_{i}=\left(\frac{\varepsilon -1}{\varepsilon +2}\right)\frac{M}{\rho }=\frac{4}{3}\pi N\alpha$$

with *M* and *ρ* being the molecular weight and density, respectively, *N* being the Avogadro number, and α being the molecule polarizability. Thus, each molecule makes a different contribution with the reacting field:

Therefore, when the soil sample is placed in the middle of two parallel plates in a capacitor configuration, the sample behaves as the dielectric, and its response is directly affected by the contribution of the physicochemical properties of the soil. By applying a voltage (V) with controlled magnitude and frequency between both terminals of the capacitor, an electric field is established (E) through the sample. The excitation signal is a sinusoidal wave with frequencies ranging from 10 Hz to 100 KHz. The electric field is directly affected by the charge separation according to their electrical charge. The frequency response allows knowing the salt and mineral content, which presents a resonance to a specific frequency. The differences in magnitude and phase between the input signal and the obtained one in the capacitor are the variables that are used to correlate the data to make an estimation of the physicochemical properties in soils.

### 2.6. Development of the System

The spectroscopy system to estimate the physicochemical variables in sugar cane soils is integrated by four sections: (a) the data signal processing spectroscopy module, (b) the capacitive sensor, (c) the controlled temperature camera, and (d) the artificial intelligence module.

**Figure 2:**
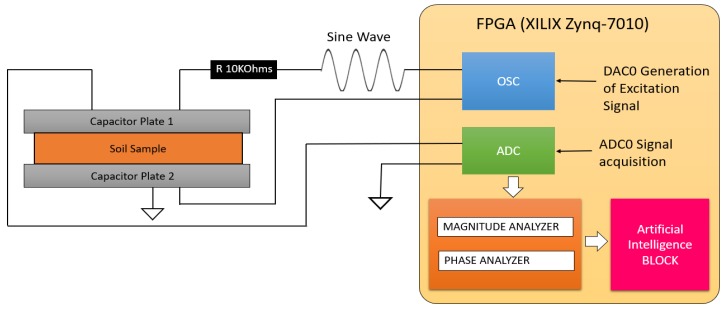
General diagram of the functioning of the capacitive sensor using the spectroscopy technique.

The module of the data signal processing spectroscopy module was developed to obtain the frequency response in terms of the magnitude and phase of a soil sample. Figure 2 shows the configuration of the electric circuit. The sensor is part of a voltage divisor and acts as an resistor-capacitance (RC) filter, which is required to know the frequency response of the sensor. The excitation signal is generated by the control module. This control module is formed by an MyRIO platform (National Instruments, Austin, TX, USA) from National Instruments that contains an FPGA XILIX Zynq-7010 and a 667 MHz dual-cortex ARM Cortex-A9 processor, which was programmed to generate the excitation signal and at the same time acquire the frequency response in magnitude and phase from the sensor. At the end of the study, it is possible to obtain the behavior of the sensor in magnitude and phase throughout the band of frequencies. The following diagram represents a distinctive pattern which allows the algorithms of artificial intelligence to estimate and quantify the content of the physicochemical variables.

The Field-programmable gate array (FPGA) generates a sine wave using a table of 2048 values. The variation of the frequency of the signal is obtained through a precise delay timer, which is executed in each one of the values of the table during the generation of the sine wave. This sine wave signal is passed through the digital/analog converter (DAC), which is connected to a 10 KOhms resistor. This resistor is connected in series with a capacitor plate 1, creating an RC circuit. The voltage output of the capacitor is acquired using an analog/digital converter (ADC), and the FPGA computes the frequency response of the sensor in real time. Figure 2 shows the general diagram of the functioning of the capacitive sensor. Equations (4) and (5) show the gain and phase computing, where Vout is the Voltage of the capacitor, and Vin is the sine wave generated by the FPGA. The Tdelay is the delay in seconds of the voltage of the capacitor with respect to the input voltage (sine wave), and Tsignal is the elapsed time of one cycle.

(4) $$Gain\;\mathrm{dB}=20log_{10}\frac{Vout}{Vin}$$

(5) $$Phase=\Phi =\frac{360{}^{\circ}\;\cdot \;Tdelay}{Tsignal}$$

**Figure 3:**
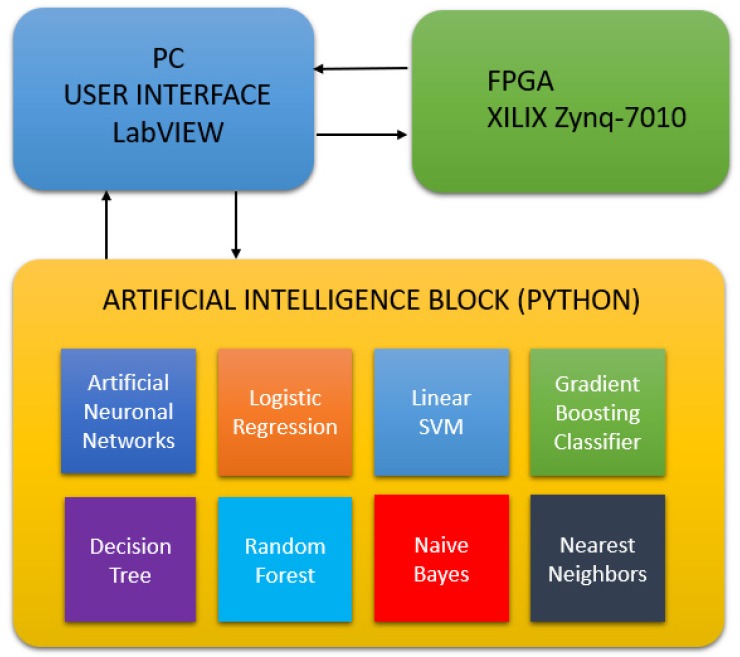
Integration of the personal computer (PC) graphical user interface (GUI) with the FPGA and the artificial intelligence block.

A graphical user interface (GUI) was designed and programmed in LabVIEW, which is connected to the FPGA and the artificial intelligence block, as it is shown in Figure 3. The frequency response of the sensor is plotted, separating the magnitude and phase values. The results are submitted to the artificial intelligence block in Python via TCP/IP. The artificial intelligence block computes different algorithms, sending the result back to the PC to be visualized in the GUI.

**Figure 4:**
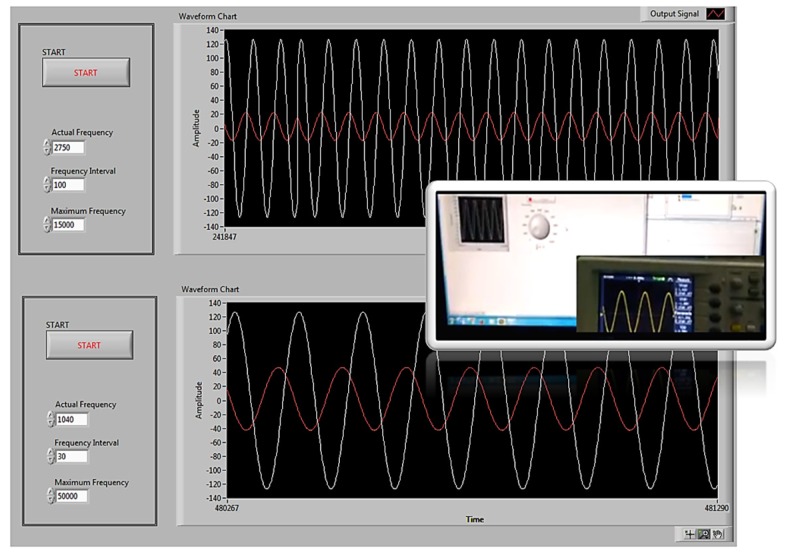
Tests of the functioning of the system in real time.

Figure 4 shows the user interface functioning. The excitation signal can be observed in white, and the response signal can be observed in red. The changes in the magnitude and phase of the signal can be appreciated when the frequency changes.

**Figure 5:**
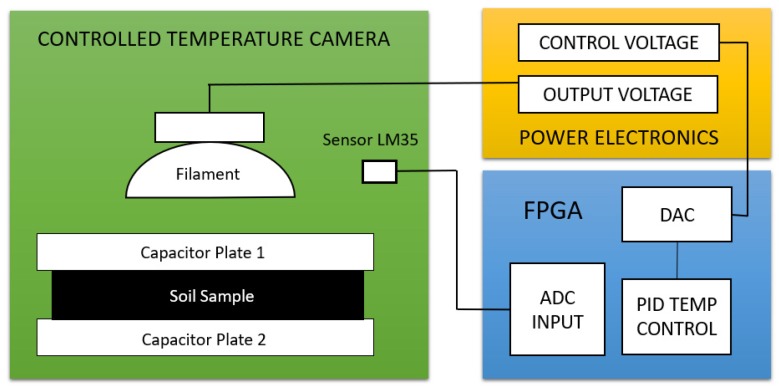
Integration of the controlled temperature camera.

In the derived equation, a constant temperature is assumed, but physicochemical properties can be affected by the temperature. Therefore, a chamber of controlled temperature was necessary, due to the temperature generating deviations in the magnitude and phase graphics of the signal. To achieve the above, a temperature sensor (LM35) was used to monitor and control the temperature in the measuring chamber; this sensor with an incandescent filament generates thermic radiation inside the camera. The voltage supplied to the filament was generated by an electronic dimmer. This dimmer requires a control voltage, which is supplied by the FPGA. The programmed Proportional Integrative Derivative (PID) temperature control, inside the FPGA, has an established set point at 30 °C as the reference point of the controller. The temperature was chosen because this is the average temperature at the locations with sugar cane crops. Figure 5 schematically shows the integration of the FPGA, the power electronics, and the controlled temperature chamber.

**Figure 6:**
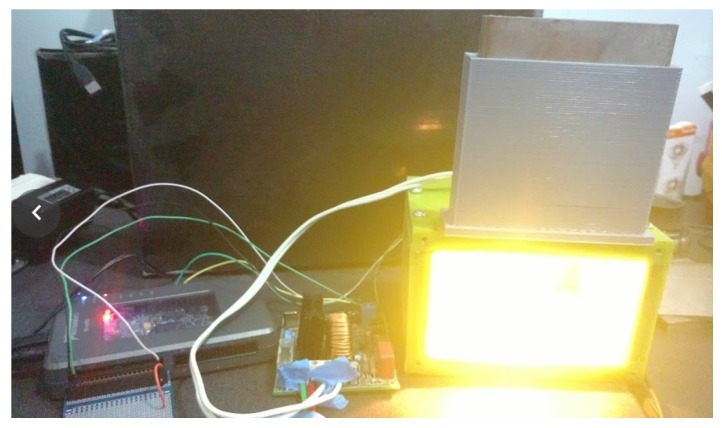
PID control of the controlled temperature camera.

Figure 6 shows a picture of the real experimental setup, with a sample placed inside the controlled temperature chamber, and the PID control running to reach the objective of 30 °C inside the chamber.

### 2.7. Sample Measurement

The experimental measurement is performed as follows. The soil sample is placed in the measurement module, filling all of the empty space between sensor electrodes. A one V pp sinusoidal wave was applied to the sample as an excitation signal. A frequency sweep was performed from 1 kHz to 100 kHz upward and downward. All of the frequencies were logarithmically spaced, and the amplitude and phase of the sensor signal were recorded. For testing the reliability of the sensor measurement, each sample was divided in eight sub-samples; all were submitted separately to the analysis, and the error measurement was calculated.

### 2.8. Artificial Intelligence for Determining Physicochemical Variables

The frequency response of the soil itself can neither determine nor quantify the physicochemical properties; thus, artificial intelligence (AI) analysis was implemented to the frequency response for determination of the physicochemical properties. Several artificial intelligence algorithms were applied for determining the physicochemical properties of the soils from the results obtained by the capacitive sensor with spectroscopy.

Algorithms from the supervised learning class were used; in particular, Artificial Neural Networks, Logistic Regression, Linear Support Vector Machine (SVM), Gradient Boosting Classifier, Decision Tree, Random Forest, Naive Bayes, and Nearest Neighbors were selected. The reason for using more than one AI algorithm is for comparing the performance and the degree of recognition for each algorithm. The Scikit-learn library was used in Python to train, analyze, and compute all of the information acquired by the sensor in real time.

**Figure 7:**
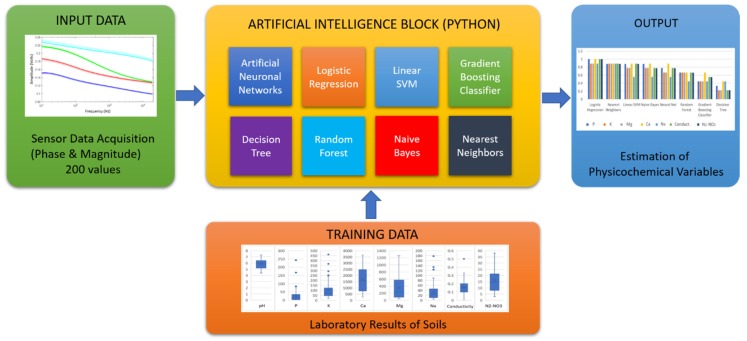
Determining psychochemical properties of soil using artificial intelligence (AI) algorithms and the sensor data.

A subset of the samples was used for training the algorithms and the rest of the sub-samples for validation. For comparison purposes, the data of the training results, the results with the test data, and the time of training were obtained for each of the algorithms. A ratio of 70/30 is usually implemented to divide the training and validating data; this ratio is chosen due to it containing enough data to accomplish the analysis. Figure 7 indicates the procedure to determine the psychochemical properties of soil using artificial intelligence algorithms. The input data acquired by the sensor is sent via transfer control protocol/internet protocol (TCP/IP) to Python, in which the artificial intelligence block is programmed.

The data from a total of 100 soil samples were used, which corresponded to the magnitude and phase data of the study of soils carried out by the capacitive sensor via spectroscopy. The input array used was an array of 200 elements (corresponding to the magnitude and frequency response of soil from 0 Hz to 100 kHz). The data used in the output, the ones with which the classifier was trained, were the physicochemical data obtained at the laboratory: pH, P, K, Ca, Mg, Na, Conductivity, and N_2_-NO_3_.

## 3. Results and Discussion

**Figure 8:**
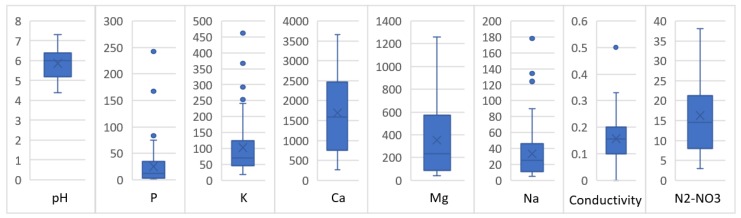
Variables statistics of the 100 soil samples (pH, P, K, Ca, Mg, Na, conductivity, and N_2_-NO_3_).

The results from the laboratory analysis served as a database used for training an AI block, as shown in Figure 8; in particular, this contained the pH, P, K, Ca, Mg, Na, conductivity, and N_2_-NO_3_ results from the analyzed soil samples.

### 3.1. Magnitude and Phase Results of the Frequency Response of the Sensor

**Figure 9:**
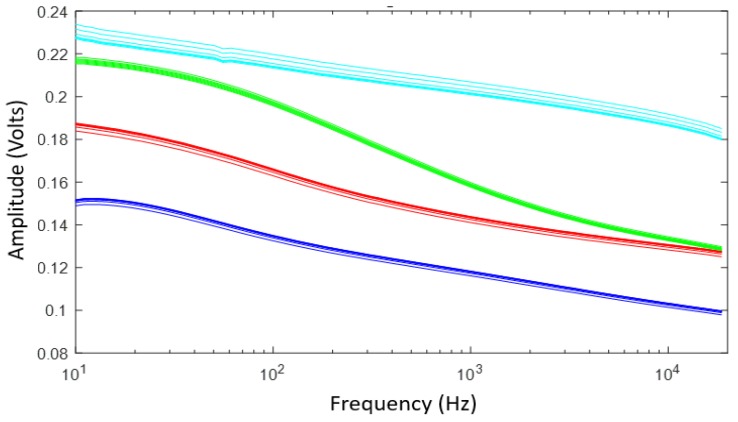
Magnitude results of the frequency response of the sensor of four soil samples and their sub-samples.

**Figure 10:**
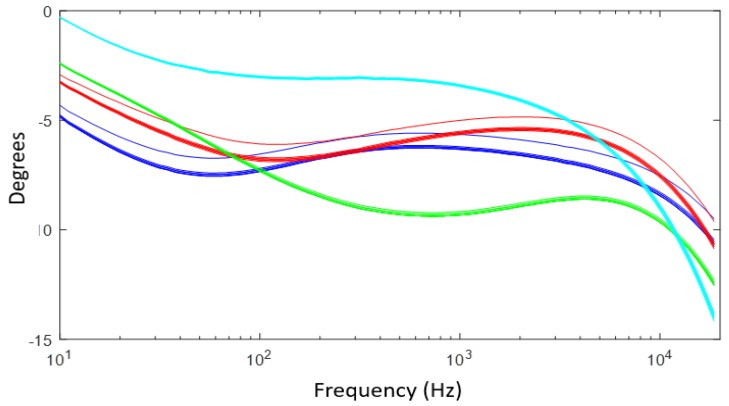
Phase results of the frequency response of the sensor of four soil samples and their sub-samples.

The frequency response of four samples in amplitude (Figure 9) and phase (Figure 10) for the most different samples, according to the laboratory measurements, was obtained. Each sample was subdivided into eight sub-samples; the sub-samples are plotted using the same color as their corresponding soil sample. As can be seen from Figure 9 and Figure 10, the frequency response behaves similarly with soils that have the same physicochemical conditions.

According to the analysis performed to the complete sample dataset, there is hysteresis when different sub-samples are measured; furthermore, the hysteresis takes different values. In the frequency range from 1 Hz to 1 KHz, the sensor presents an 8.3% difference, and from 1 KHz to 100 KHz, it presents a difference of 3.61%. The analysis of repeatability of the 100 soil samples computed a mean quadratic error of 4.96%, which means that the repeatability of the sensor is roughly 95%.

### 3.2. Sensor Response as a Function of Temperature

**Figure 11:**
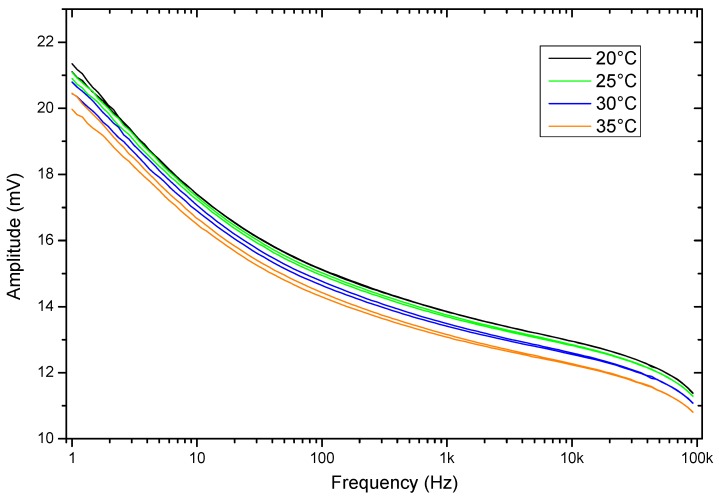
Magnitude results of the sensor response as a function of temperature.

**Figure 12:**
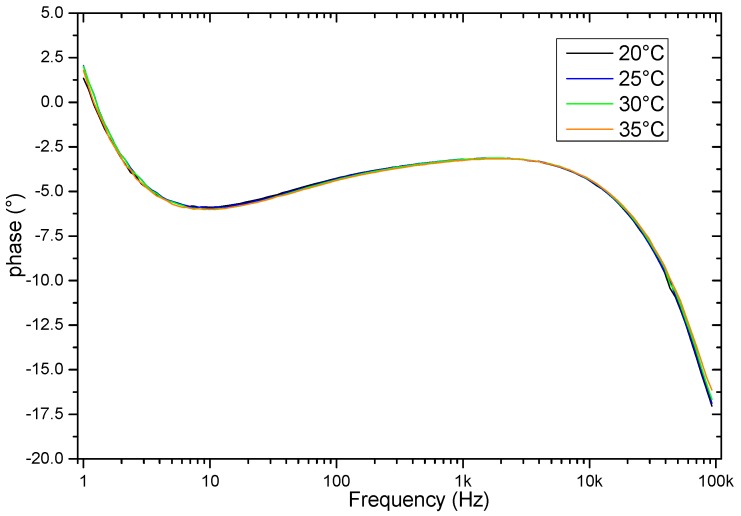
Phase results of the sensor response as a function of temperature.

The evaluation of the behavior when there are temperature variations is also relevant. Therefore, to investigate the effects of temperature on the sample frequency scanning, the measurements were performed in the temperature range from 20 °C to 35 °C. Figure 11 and Figure 12 show the results in these temperature variations of the frequency response of the sensor that contains a soil sample. The results indicate variations mainly in magnitude values, indicating that there is a proportional amplitude reduction of the signal when the temperature increases.

**Figure 13:**
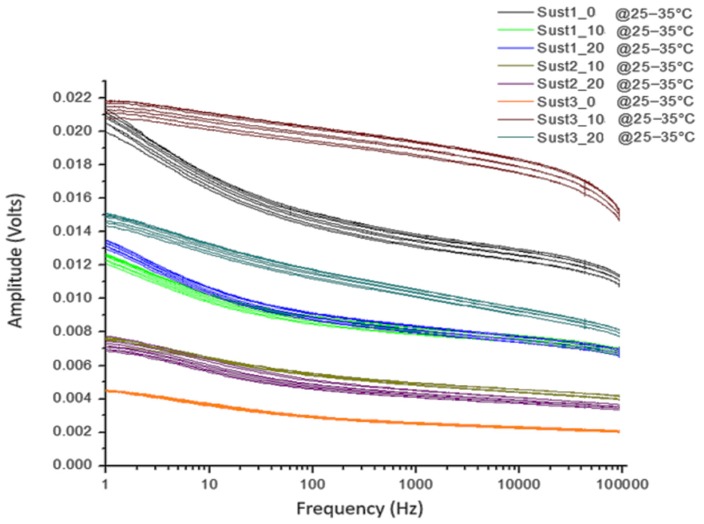
Amplitude frequency response as a function of the temperature for eight samples.

**Figure 14:**
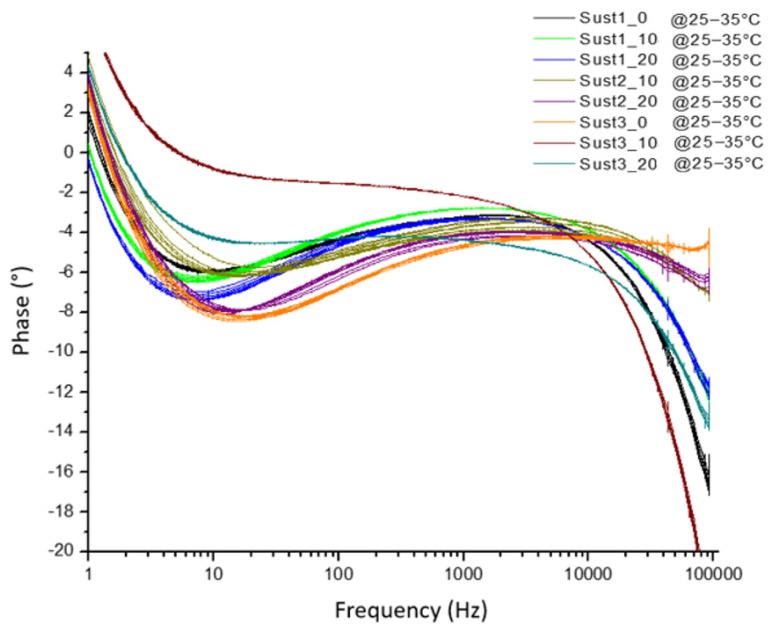
Phase frequency response as a function of the temperature for eight samples.

A group of eight soil samples was used to validate the frequency response of the sensor when there is a variation in temperature. For each soil sample, the temperature was set in the range from 25 °C to 35 °C in steps of 2.5 °C. As Figure 13 and Figure 14 show, the amplitude is reduced proportionally to the increment of temperature. The eight soil samples were clustered by a specific color, and each cluster contained the information of the frequency response in different temperatures. In all of the cases, the maximum amplitude of the frequency response of the sensor corresponded to the minimum temperature.

### 3.3. Artificial Intelligence Analysis

**Figure 15:**
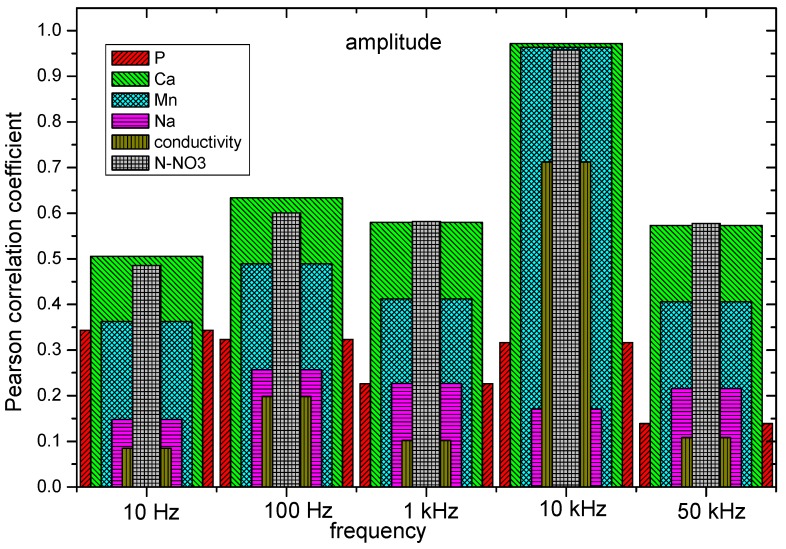
Pearson correlation coefficient of P, Ca, Mn, Na, conductivity, and N_2_-NO_3_ with respect to the amplitude in the range of frequencies of 10 Hz, 100 Hz, 1 KHz, 10 KHz, and 50 KHz.

**Figure 16:**
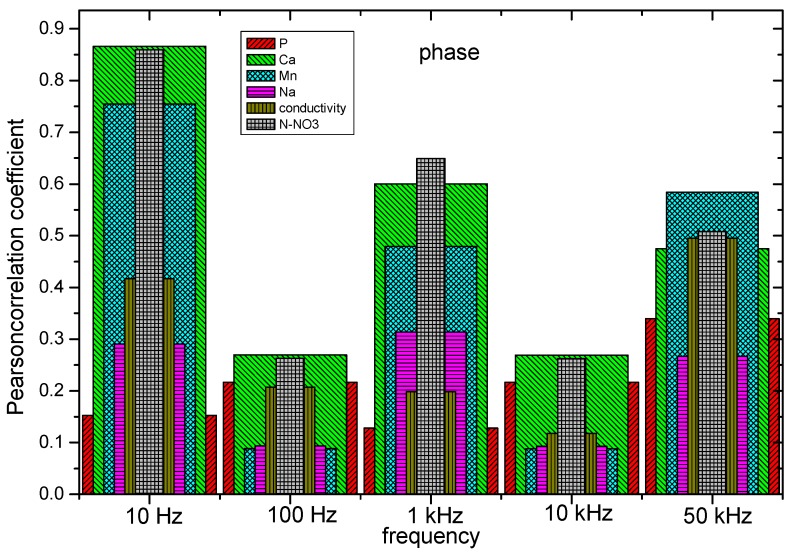
Pearson correlation coefficient of P, Ca, Mn, Na, conductivity, and N_2_-NO_3_ with respect to the phase in the range of frequencies of 10 Hz, 100 Hz, 1 KHz, 10 KHz, and 50 KHz.

As the first step in the signal analysis, the Pearson correlation coefficient was obtained for the different physiochemical properties with respect to five frequency intervals in amplitude and phase. The frequencies ranges were selected by decades. Results from the calculated correlation coefficient show a direct relationship between the physicochemical properties of soils with respect to the magnitude and phase of the sensor. From Figure 15 and Figure 16, the correlation of the pH, P, K, Ca, Mn, Na, conductivity, and N_2_-NO_3_ with respect to the magnitude and phase of the frequency response of the sensor is shown. The results of magnitude and phase for the frequency response of the sensor of the 100 soil samples were clustered in 10 groups: five groups for amplitude, and five for phase.

The correlation coefficient results indicate a hierarchy pattern between magnitude and phase with respect to the psychochemical analysis of soil. It can be observed that the psychochemical variables (Na, Mn, K, Ca, P, pH, conductivity, and N-NO_3_) have a hierarchy pattern that is different between themselves with respect to the magnitude and phase components of the sensor. Therefore, each variable has a characteristic response in the determined magnitude and phase points of the sensor.

**Figure 17:**
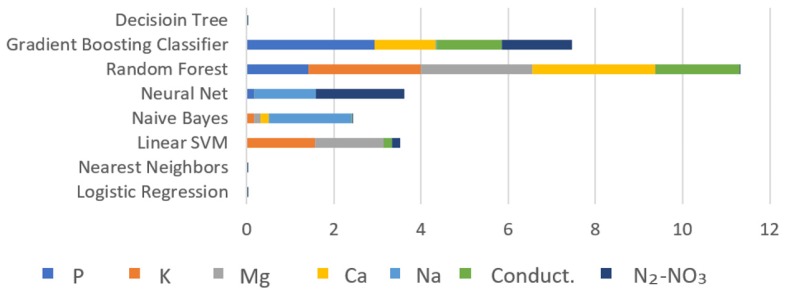
Training times (seconds) comparison among classifiers.

By using the frequency response of the soil, a complex set of information is generated. Despite there being differences in the amplitude or phase spectra when the temperature, moisture, or chemical content varies, the differences cannot give useful information regarding the soil state. Amplitude and phase spectra were subject to different AI algorithms, which were written to predict the chemical composition of the soil. The implementation of the AI algorithms was carried out to obtain the estimation of the psychochemical variables of sugar cane soil. Figure 17 shows a graphic with the training times of classification of the P, K, Mg, Ca, Na, conductivity, and N_2_-NO_3_ using the AI algorithms of Decision Tree, Gradient Boosting Classifier, Random Forest, Neuronal Networks, Naive Bayes, Linear SVM, Nearest Neighbors, and Logistic Regression. The results indicate that Logistic Regression and Nearest Neighbors were trained faster than the rest of the algorithms.

**Figure 18:**
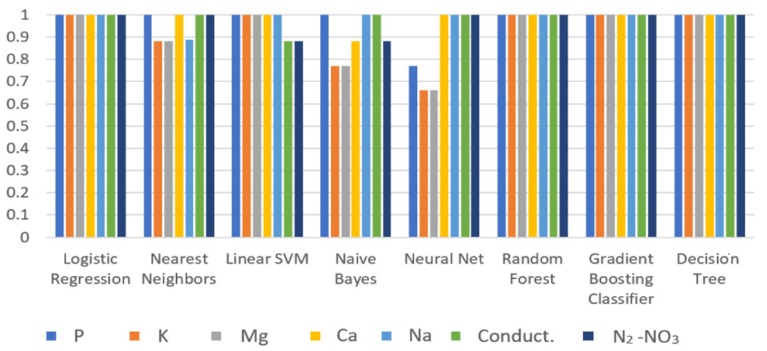
Train score.

The 70% of the data were used to train the AI algorithms. Figure 18 shows the punctuation of the training process using pH, P, K, Ca, Mg, Na, conductivity, and N_2_-NO_3_ values for all of the AI algorithms that were implemented. It should be noted that the correlation coefficient of one was reached with the Logistic Regression, Random Forest, Gradient Boosting Classifier, and Decision Tree algorithms.

**Figure 19:**
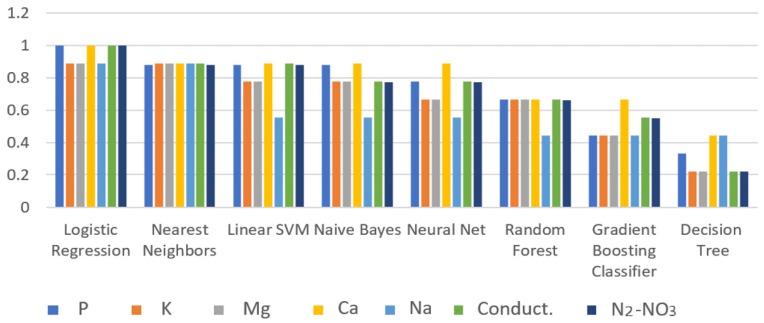
Test score comparison for all of the classifiers.

In order to validate the efficiency of the trained algorithms, 30% of the missing data was used to test the trained algorithms. Figure 19 shows the score obtained from the classification process using the magnitude and phase variables of the study of spectroscopy and the physicochemical data of the laboratory for: pH, P, K, Ca, Mg, Na, conductivity, and N_2_-NO_3_. Despite the Decision Tree reaching a correlation coefficient of one during the training process, this algorithm had the worst punctuation in the classification, whereas Logistic Regression and Nearest Neighbors obtained the highest scores. Only the P, Ca, conductivity, and N_2_-NO_3_ obtained a correlation coefficient of one; for the rest of the physicochemical variables, the highest correlation value was 0.9.

Through all of the experiments that were developed, it was possible to confirm that the use of classifiers such as Logistic Regression, Nearest Neighbors, and Linear SVM were reliable algorithms for the recognition and estimation of the desired variables. The correlation coefficient reached by using Logistic Regression and Nearest Neighbors was more than 0.9. The obtained results from the AI algorithms could give a rough estimation of the physicochemical state of the soil under study. Considering the technique used in this work, the accuracy obtained is good enough for serving as an indicator of the soil state.

## 4. Conclusions

The development of a new system that is capable of estimating the phosphorus, potassium, magnesium, calcium, sodium, conductivity, and nitrogen–nitrate in soils by the implementation of low-frequency spectroscopy techniques and artificial intelligence was achieved. The obtained results indicate that the instrument presents repeatability, and the artificial intelligence algorithms such as Logistic Regression and Nearest Neighbor can obtain results with an accuracy above 90% in its recognition. This makes the instrument appropriate for obtaining an adequate estimation of some of the most relevant physicochemical properties in soils through a prompt, simple, and low-cost means. In this study, these algorithms were successfully implemented in an FPGA-based system, which served at the same time as the signal acquisition system. The system that has been presented here is a useful alternative for people with little technical experience who require an analysis of the soil to produce more or better sugar cane; furthermore, it could also be helpful to improve the decisions made for its restoration.

For future improvement of the proposed system, a cross-validation using more than one classifier could be implemented. In order to determine universal soil properties, further studies are needed using different soil samples. Even so, the presented results show the suitability of the proposed system as a soil physicochemical indicator.

## References

[1] Ortiz Bernad I., Sanz García J., Dorado Valiño M., Villar Fernández S. (2007). Técnicas de Recuperación de Suelos Contaminados.

[2] Robert M. (2002). Captura de Carbono en Los Suelos Para un Mejor Manejo de la Tierra.

[3] European Commission (2006). Thematic Strategy for Soil Protection 2006.

[4] USDA Natural Resources Conservation Service. http://www.nrcs.usda.gov/wps/portal/nrcs/detail/soils/use.

[5] Hernández A.J., Pastor J. (2008). La restauración en sistemas con suelos degradados: Estudio de casos en agroecosistemas mediterráneos y taludes de carretera. Contaminación de Suelos. Tecnologías Para su Recuperación.

[6] Altieri M., Hecht S., Liebman M., Magdoff F., Norgaard R., Sikor T.O. (1999). Agroecología: Bases científicas para una agricultura sustentable.

[7] Glissman S.R. (2002). Agroecología. Procesos ecológicos en Agricultura Sostenible.

[8] Garrabou S.R., González de Molina M. (2010). La Reposición de la Fertilidad en los Sistemas Agrarios Tradicionales.

[9] FAO Soils Portal. http://www.fao.org/soils-portal/soil-survey/clasificacion-de-suelos/sistemas-numericos/propiedades-quimicas/es/.

[10] Oldeman L.R. (2000). Impact of Soil Degradation: A Global Scenario.

[11] Eckert S., Hüsler F., Liniger H., Hodel E. (2015). Trend analysis of MODIS NDVI time series for detecting land degradation and regeneration in Mongolia. J. Arid Environ..

[12] Adamchuk V.I., Hummel J.W., Morgan M.T., Upadhyaya S.K. (2004). On-the-go soil sensors for precision agriculture. Comput. Electron. Agric..

[13] Serrano J.M., Peça J.O., Marques da Silva J.R., Shaidian S. (2010). Mapping soil and pasture variability with an electromagnetic induction sensor. Comput. Electron. Agric..

[14] Lasia A. (1999). Electrochemical Impedance Spectroscopy and its Applications. Mod. Asp. Electrochem..

[15] Pandey G., Kumar R., Weber R.J. (2014). A Low RF-Band Impedance Spectroscopy Based Sensor for In Situ, Wireless Soil Sensing. IEEE Sens. J..

[16] Kodaira M., Shibusawa S. (2013). Using a mobile real-time soil visible-near infrared sensor for high resolution soil property mapping. Geoderma.

[17] Aqeel U.R., Abbasi A.Z., Islam N., Shaikh Z.A. (2014). A review of wireless sensors and networks’ applications in agriculture. Comput. Stand. Interfaces.

[18] Nocita M., Stevens A., Noon C., Van Wesemael B. (2013). Prediction of soil organic carbon for different levels of soil moisture using Vis-NIR spectroscopy. Geoderma.

[19] FAO (2015). World Reference Base for Soil Resources, in International Soil Classification System for Naming Soils and Creating Legends for Soil Maps.

[20] USEPA (U.S. Environmental Protection Agency) (2014). SESDPROC-300-R3 Soil Sampling.

[21] Willits C.O. (1951). Methods for Determination of Moisture-Oven Drying. Anal. Chem..

[22] Walkley A., Black I.A. (1934). An Examination of the Degtjareff Method for Determining Soil Organic Matter and a Proposed Modification of the Chromic Acid Titration Method. Soil Sci..

[23] Pleysier J.L., Juo A.S.R. (1980). A single-extraction method using silver-thiourea for measuring exchangeable cations and effective cec in soils with variable charges. Soil Sci..

[24] Bremner J.M. (1960). Determination of nitrogen in soil by the Kjeldahl method. J. Agric. Sci..

[25] Menon R.G., Chien S.H. (1995). Soil testing for available phosphorus in soils where phosphate rock-based fertilizers are used. Fertil. Res..

[26] Rodrigues A.L., Molina R., Bendassolli J.A., Ochueza Trivelin P.C. (2008). Organic sulfur oxidation to sulfate in soil Samples for total sulfur determination by turbidimetry. Rev. Bras. Cienc. Solo.

[27] Burt R., USDA, Soil Survey Staff (2014). 2014 Soil Survey Staff. Kellogg Soil Survey Laboratory Methods Manual.

